# Synthesis, Antimicrobial, and Antioxidant Activities of Chalcogen-Containing Nitrone Derivatives from (*R*)-citronellal

**DOI:** 10.3390/medicines4020039

**Published:** 2017-06-10

**Authors:** Mariana C. Ferraz, Renata A. Mano, Daniela H. Oliveira, Darla S. V. Maia, Wladimir P. Silva, Lucielli Savegnago, Eder J. Lenardão, Raquel G. Jacob

**Affiliations:** 1 Laboratório de Síntese Orgânica Limpa, LASOL, Universidade Federal de Pelotas, UFPel, Pelotas, RS 96010-900, Brazil; mariana.c.f@hotmail.com (M.C.F.); renata-mano@hotmail.com (R.A.M.); dani.hartwig@gmail.com (D.H.O.); 2 Faculdade de Agronomia Eliseu Maciel, DCTA, Universidade Federal de Pelotas, UFPel, Pelotas, RS 96010-900, Brazil; wladimir.padilha2011@gmail.com (D.S.V.M.); wladimir@pq.cnpq.br (W.P.S.); 3 Centro de Desenvolvimento Tecnológico, Unidade Biotecnologia, Universidade Federal de Pelotas, UFPel, Pelotas, RS 96010-900, Brazil; lucielli@ufpel.edu.br

**Keywords:** citronellal, organochalcogen, foodborne bacteria, antioxidant, antibacterial

## Abstract

**Background:** The main constituents of *Cymbopogonnardus* (L) Rendle and C. *citratus* (DC) Stapfessential oils are (*R*)-citronellal and citral, respectively. Organochalcogen compounds can boost the biological activities of natural products. **Methods:** Several chalcogen-containing nitrones derived from (*R*)-citronellal and citral were prepared and evaluated for their antimicrobial and antioxidant activities. The antimicrobial activity was evaluated by the disc diffusion test and the antioxidant properties were evaluated in vitro by DPPH (1,1-diphenyl-2-picryl-hydrazyl), ABTS (2,2′-azino-bis(3-ethylbenzthiazoline-6-sulfonic acid), and FRAP (ferric ion reducing antioxidant power) assays. **Results:** In the antimicrobial assay, (*E*)-*N*,3,7-trimethyl-3-(phenylthio)oct-6-en-1-imine oxide **5c** exhibited halos between 21.5 mm (*Escherichia coli* O157:H7) and 26.0 mm (*Listeria monocytogenes*), while (*E*)-*N*,3,7-trimethyloct-6-en-1-imine oxide **5d** presented halos between 22.5 mm (*E. coli* O157:H7) and 31.0 mm (*L. monocytogenes*). (*E*)-*N*,3,7-Trimethyl-2-(phenylthio)oct-6-en-1-imine oxide **5a** showed the lowest minimal inhibitory concentration (MIC) value against *Bacillus cereus* (0.48 mM), and **5c** was the most potent bactericide, with a minimal bactericidal concentration (MBC) of 0.52 mM for *E. coli* O157:H7. In the antioxidant assays, **5c**, **5d**, and **10** ((*E*)-3,7-dimethyl-2-(phenylselanyl)oct-6-enal oxime) were the most actives in the DPPH, ABTS, and FRAP assays, respectively. **Conclusions:** The presence of a phenylthio group in the nitrone increases its antimicrobial activity against Gram-positive and Gram-negative foodborne pathogens in the disk diffusion test and the antioxidant activity in vitro.

## 1. Introduction

Oxidative and microbial changes are among the main causes of loss of quality and value of food products and infections and intoxication caused by pathogenic microorganisms bring risk to the consumer health [1]. To minimize or avoid these problems, antioxidants and preservatives are added for microbiological control. The search for a better understanding of the genetic mechanisms of microbial resistance and studies on new antimicrobials and antioxidants—synthetic, semi-synthetic, or natural ones—are of great interest [2].

In this context, many studies looking for the prospection and identification of naturally-occurring bioactive compounds—such as essential oils (EOs) and their constituents—were recently described. A very versatile natural compound is citronellal, which is a monoterpene predominantly formed by the secondary metabolism of plants, usually found as a non-racemic mixture of *R* and *S* enantiomers. Citronellal is present in EOs of various plant species, being the main component of the *Cymbopogon nardus* EO, where (+)-(*R*)-citronellal is the only enantiomer found [3]. Several biological properties are associated with citronellal, including significant antioxidant and antimicrobial activities. Nevertheless, there are a number of limitations in the use of EOs or their constituents as food additives, which range from the low stability, interaction with ingredients, and other additives to the impact on the sensory characteristics of the product [4].

The development of new preservatives is directly linked to the economic importance of the food’s spoilage. The chemical modification of natural products has attracted the attention of many research groups, which aims to improve their original biological activities [5,6,7,8]. Recent studies have shown that semi-synthetic compounds functionalized with organochalcogen groups (sulfur and selenium) and nitrones could be an alternative to the synthetic additives currently used in food and pharmaceutical industries [8].

Organoselenium and organosulfur substrates have shown to be ideal to increase several biological and pharmaceutical activities, such as antibacterial, antifungal [9], and antioxidant [10]. This enhancing can be attributed, at least in part, to the ability of selenium and sulfur to stabilize free radicals [11].

Nitrones are molecules that contain nitrogen and oxygen heteroatoms and their 1,3-dipolar structure favors their participation in the synthesis of various biologically active nitrogen-containing substances [12,13]. Nitrone derivatives are industrially used in numerous applications, including as binders, antifungals, antimicrobials, and as starting materials in the synthesis of important organic compounds, such as oxaziridines, isoxazolidines, hydroxylamines, among others [14,15,16,17,18].

Therefore, based on the previous considerations and in continuation to our studies on the synthesis of semi-synthetic organochalcogen compounds, the objective of this work was to combine the bioactive properties of nitrone and the organochalcogen group with those of citronellal, a natural terpenoid, to obtain new multifunctional compounds. The new semi-synthetic compounds designed by molecular hybridization were evaluated for their antimicrobial and antioxidant activities in vitro.

## 2. Materials and Methods

### 2.1. Oil Material

Essential oil of citronella (*C. nardus* (L.) Rendle) was commercial product, produced in southern Brazil (Pólo Oleoquímico de Três Passos-RS). (*R*)-Citronellal was isolated from the essential oil by column chromatography using silicagel as a stationary phase and a solution of hexane/ethyl acetate (99:1) as the eluent. Citral (a 1:1 mixture of neral and geranial) and the other reagents were purchased from Aldrich (St. Louis, MO, USA).

### 2.2. Synthesis of α-phenylselanyl citronellal **3a**, α-phenylthio citronellal **3b**, and β-phenylthio citronellal **8**

**Figure 1:**
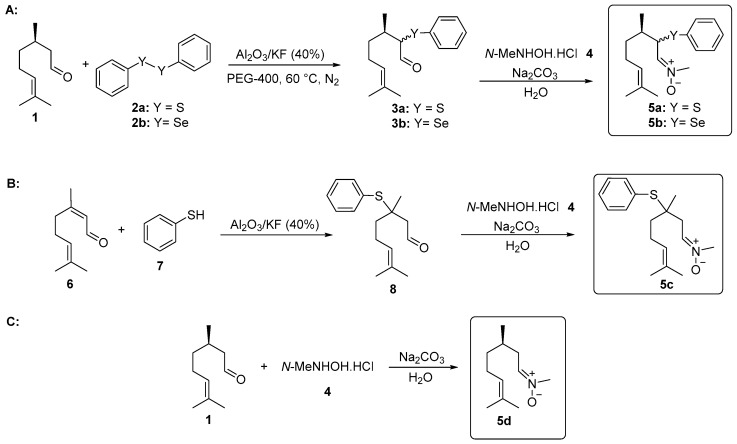
Synthesis of nitrones derived from citronellal. (A) α-Phenylselanyl citronellal **3a**, α-phenylthio citronellal **3b** and nitrones **5a** and **5b**; (B) β-Phenylthio citronellal **8** and nitrone **5c**; (C) Nitrone **5d**.

The synthesis of *α*-phenylchalcogencitronellal **3a–b** was performed according to the methodology developed by Nazari and Movassagh [19], with modifications. In a 25 mL vial was added (*R*)-citronellal (**1**, 0.308 g, 2 mmol), diphenyl disulfide (**2a**, 1.5 mmol), or diphenyl diselenide (**2b**, 2 mmol) and PEG-400 (4.0 mL) under N_2_ atmosphere. Then, Al_2_O_3_/KF (40%) (0.324 g, 1.5 mmol) was added and the temperature was slowly increased to 60 °C. The progress of the reaction was monitored using thin layer chromatography (TLC) and after 22 h, compounds **3a–b** were isolated and characterized (Figure 1A).

The synthesis of *β*-phenylchalcogencitronellal **8** was performed according to previously described by our group [20]. In a test tube was added citral (**6**, 0.304 g, 2 mmol), benzenethiol (**7**, 0.352 g, 2.4 mmol) and Al_2_O_3_/KF (40%) (0.140 g, 0.65 mmol) under magnetic stirring at room temperature. The progress of the reaction was monitored using thin layer chromatography (TLC) and after 24 h, compound **8** was isolated and characterized (Figure 1B).

### 2.3. General Procedure for the Synthesis of Nitrones **5a–d** Derived from Citronellal

The functionalized nitrones **5** were prepared using a synthetic route adapted from Isager et al. [21]: In a 25 mL vial, aldehyde **1**, **3a–b**, or **8** (0.5 mmol); *N*-methyl-hydroxylamine hydrochloride (**4**, 0.084 g, 1 mmol); and water (2 mL) as the solvent were combined. The mixture was stirred at room temperature for 30 min. Then, a 1M solution of sodium carbonate (Na_2_CO_3_) (1.0 mL) was added and the stirring was continued for additional 24 h. Compound **5a** was purified by preparative chromatographic plate (silica gel) and compounds **5b–d** were isolated by column chromatography using neutral alumina as a stationary phase and a solution of hexanes/ethyl acetate (90:10) as the eluent (Figure 1A–C).

### 2.4. Synthesis of the Selenium-Containing Oxime **10**

**Figure 2:**
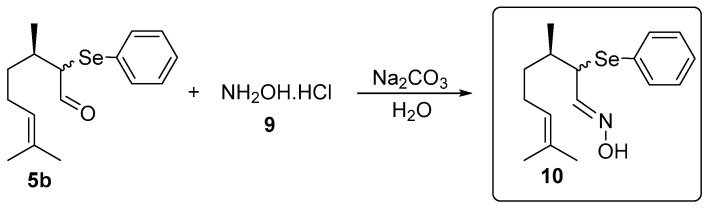
Synthesis of (3*R*,*E*)-3,7-dimethyl-2-(phenylselanyl)oct-6-enal oxime **10**.

The oxime **10** was prepared using a synthetic route adapted from Isager et al. [21]: In a 25 mL vial *α*-phenylselenocitronellal (**5b**, 0.156 g, 0.5 mmol), hydroxylamine hydrochloride (**9**, 0.069 g, 1 mmol) and water (2 mL) as the solvent were combined. After stirring for 30 min at room temperature, 0.5 mL of an aqueous solution of Na_2_CO_3_ (0.027 g, 0.26 mmol) was added and the stirring was continued for additional 22 h. After this time, the oxime **10** was isolated by column chromatography using silica gel as a stationary phase and a solution of hexanes/ethyl acetate (95:5) as the eluent (Figure 2).

### 2.5. Bacterial Strains

To evaluate the antimicrobial potential of the synthesized compounds, the following reference strains of bacteria, that are economically important in foods, were employed: Gram-positive *Listeria monocytogenes* ATCC 7644, *Staphylococcus aureus* ATCC 25923, and *Bacillus cereus* ATCC 11778; Gram-negative *Salmonella* Typhimurium ATCC 14028, *Escherichia coli* O157: H7 NCTC 12900 and the clinically important *Pseudomonas aeruginosa* ATCC 15442.

The antimicrobial activity assay using the disk diffusion test was realized using the methodology that is recommended by the Clinical Laboratory Standards Institute (CLSI) [22]. The determination of the minimum inhibitory concentration (MIC) and minimum bactericidal concentration (MBC) was performed using the macro dilution tube method, in accordance with Rota et al. [23], with modifications. Details of these assays are provided in the Supplemental Material.

### 2.6. Antioxidant Activity Assays

The antioxidant properties of the synthesized compounds (**5a–d** and **10**) were evaluated by three different methods in vitro: DPPH (1,1-diphenyl-2-picryl-hydrazyl) and ABTS^+^ (2,2′-azino-bis(3-ethylbenzthiazoline-6-sulfonic acid) radical scavenging ability and ferric ion reducing antioxidant power (FRAP). All drugs were dissolved in dimethyl sulfoxide (DMSO) at concentrations of 1–500 µM. These assays were performed according to the literature and details of these experiments are described elsewhere (see the ‘Results and Discussion’ section for the references).

### 2.7. In Vitro Toxicity

The assay used to measure the activity of δ-ALA-D quantifies the products of the enzymatic action in two molecules, δ-aminolevulinic acid (ALA), and porphobilinogen (PBG), which after reaction with the Ehrlich’s solution, generates a red-pinkish color [24,25]. The activity of δ-ALA-D in the presence of compounds **5a–d** and **10** at different concentrations (10–500 μM) was determined according to the method described by Sassa [24]. Detailed information is in the Supplemental Material.

## 3. Results and Discussion

### 3.1. Chemistry

**Figure 3:**
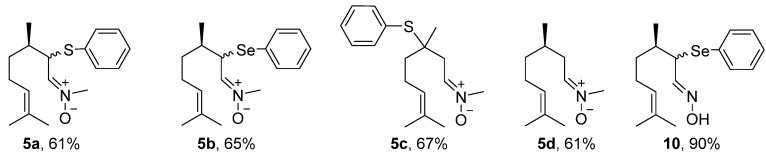
Yields of nitrones derived from citronellal **5a–d** and α-phenylselanyl oxime **10**.

The first step in the synthesis of the chalcogen-containing nitrones **5a–c** and the α-phenylselenoxime **10**, derived from citronellal **1** involves the preparation of the key intermediates α- and β-phenylchalcogen citronellal **3a–b** and **8**. For **3a–b**, a synthetic route described by Nazari and Movassagh [19] was used, while the β-phenylthio citronellal **8** was prepared using our previously reported procedure [20]. With the starting materials **3a–b** and **8** in hands, they were subjected to the reaction with *N*-methyl-hydroxylamine hydrochloride **4** to prepare **5a–c**; α-phenylselanyl citronellal **3b** was also reacted with hydroxylamine hydrochloride **9** to prepare the oxime **10**. Products were purified by preparative chromatographic plate (**5a**), column chromatography on neutral alumina (**5b** and **5c**), or using silicagel (**10**). The nitrone derivative from citronellal **5d** was prepared directly from the freshly purified aldehyde **1** and purified by column chromatography using neutral alumina. The structures and yields of the five prepared compounds are presented in Figure 3. In general, products were obtained in good yields (61–90%) and they showed a good stability after purification, being characterized by ^1^H and ^13^C-NMR and mass spectrometry. Figures of NMR spectra (^1^H and ^13^C) of compounds **5a–d** are presented in the Supplementary Material. The NMR spectra of nitrones **5a** (Figures S1 and S2), **5b** (Figures S3 and S4), **5c** (Figures S5 and S6), **5d** (Figures S7 and S8) and oxime **10** (Figures S9 and S10) are in accordance with those expected for the compounds. Compounds **5a–b** and **10** were obtained as a 1:1 mixture of diasteroisomers, once a second stereocenter is generated in the precursor β-phenylchalcogen citronellal **3a** and **3b**. Because it was not separable by the chromatographic methods employed by us, the mixture of isomers was used in the bioassays.

### 3.2. Antimicrobial Activity

In the first set of experiments, the antimicrobial activity of nitrones **5a–d** and α-phenylselenoxime **10**, derived from citronellal, was evaluated in vitro using the agar disk diffusion test. The strains *L. monocytogenes*, *S. aureus, B. cereus*, *S.* Typhimurium, *E. coli* O157:H7, and *P. aeruginosa* were qualitatively and quantitatively assessed for the presence or absence of inhibition zones (zone diameters) (Table 1), the minimum inhibitory concentration (MIC) and the minimum bactericidal concentration (MBC) (Table 2).

As it can be seen on Table 1, nitrones **5c** (containing a phenylthio group in the β-position) and **5d** (without a chalcogen) and the seleno-containing oxime **10** generated inhibition zones for Gram-positive bacteria comparable to the control antibiotic streptomycin. Studies suggest that Gram-positive bacteria are more sensitive to EOs and their components than Gram-negative ones. This behavior appears to be related to the presence of the outer cell membrane in Gram-negative bacteria, which gives greater protection, making them more difficult to be inhibited [26]. Compounds **5c** and **5d** also showed a good antimicrobial potential for Gram-negative bacteria, with inhibition halos greater than those of streptomycin. These results are indicative that the chemical modification of the constituents of plants’ EOs can generate compounds with an improved antibacterial activity.

The high antimicrobial activity of compounds **5c** and **5d** were confirmed by their MIC and MBC values (Table 2), which were between 0.52 mM and 0.69 mM for compound **5c** and between 1.13 mM and 1.26 mM for **5d** in all the tested bacteria. Interestingly, compound **5a**, having a phenylthio group in the α-position, inhibited *B. cereus* selectively, presenting MIC and MBC values of 0.48 mM and 0.59 mM, respectively. The antibacterial activity of 3-(*p*-chlorophenyl)thio citronellal, a close-related compound to **3a**, was recently studied by us [27] and it showed inhibitory activity against Gram-positive *L. monocytogenes* and *S. aureus* (MIC of 2.1 mM) and Gram-negative bacteria *S.* Typhimurium, *S. dysenteriae, E. coli*, and *P. fluorescens* (MIC of 4.2 mM).

As we can see from the MIC and MBC values in Table 2, there is a clear improvement in the antimicrobial activity when an organosulfur and a nitrone group are present in the (*R*)-citronellal scaffold. The biological activities of these classes of compounds include antioxidant, anti-inflammatory, and antiviral ones; however, little information exists about the antimicrobial activity of compounds containing both organochalcogen and nitrone groups in the same molecule.

### 3.3. Antioxidant Activity

To verify the effect of the presence of the organochalcogen and nitrone moieties in the new molecules, the antioxidant activity of the nitrones derived from citronellal **5a–d** (Figure 1) and α-phenylselenoxime citronellal **10** (Figure 2) was evaluated by different methods in vitro.

As can be seen in Table 3, only compounds **5a** and **5c**—which contain the organosulfur moiety—have demonstrated DPPH radical scavenging activity. The selenium-containing nitrone **5b**, the nitrone **5d**, without chalcogen, and the selenoxime **10** did not present effect in the DPPH test (results not presented).

Table 4 shows the outcomes in the ABTS radical scavenging activity of compounds **5a–d** and **10**. In this assay, α-phenylchalcogen nitrones **5a** and **5b** and the α-phenylselenoxime **10** were the most active ones, with **10** presenting the best result (IC_50_ of 25 μM). The IC_50_ for α-phenylchalcogen nitrones **5a** and **5b** were of 297 and 315.3 μM respectively, while β-phenylthio nitrone **5c** showed an IC_50_ value of 419.30 μM. This finding indicates that the proximity of the chalcogen atom to the nitrone or oxime groups affects the ABTS radical scavenging ability.

DPPH and ABTS are the most common spectrophotometric methods to determine the antioxidant activity of organic compounds, because they can directly react with the antioxidant species [28]. The principle of the DPPH assay is a single electron transfer (SET) reaction and a hydrogen-atom removal [29], while the ABTS assay is based only on a SET [30].

Several reports in the literature have demonstrated that the antioxidant activity might be correlated to the reducing power of a compound [31,32,33]. Thus, based on this evidence, the FRAP assay was used to determine the reducing power of compounds **5a–d** and **10** to clarify the relationship between the antioxidant effect and the reducing power (Table 5).

Among the tested compounds, **5d** was the most effective in the FRAP test, with a concentration-dependent reducing power. In addition, our results revealed that all the (*R*)-citronellal derivatives presented a high ferric reducing ability.

The antioxidant and antidepressant-like activities of the semi-synthetic α-phenylselenocitronellal **3b** and the natural terpenoid (*R*)-citronellal **1** were previously evaluated by Victória et al. [9] and both were inactive in the DPPH and ABTS assays. In the FRAP assay, **3b** presented significant reducing power at concentrations equal and higher than 500 mM, while **1** did not present any effect at the same concentrations [9]. Taken together, these findings and the results of Table 3 and Table 4 indicate that the presence of the nitrone and oxime groups are crucial for the radical scavenging ability of the citronellal derivatives, while the aldehyde itself is not active, even when a chalcogen group is present.

### 3.4. In Vitro Toxicity

Based on the antimicrobial and antioxidant activities displayed by the semi-synthetic citronellal-based compounds **5a–d** and **10** and searching for future technological applications in food preservation, their acute toxicity was investigated in mice’s tissues of liver, kidney, and brain (Table 6). The enzymatic activity of δ-Ala-D was evaluated at different concentrations (10, 50, 100, and 500 μM) of compounds **5a–d** and **10** and no decrease was observed for **5a**, **5c**, **5d**, and **10** in all the tested concentrations. This suggests the absence of acute toxicity in vitro. However, nitrone **5b**, containing a phenylselanyl group at the α-position of the nitrone, inhibit the δ-Ala-D activity at all concentrations in the liver and at 100 and 500 μM in kidney. This suggests an acute toxicity of **5b** in vitro.

## 4. Conclusions

A new class of chalcogen-containing nitrones derivatives from (*R*)-citronellal, obtained from a renewable source, *C. nardus* (L.) Rendle essential oil, was synthesized and evaluated for their antimicrobial and antioxidant activities in vitro. From this study, it can be concluded that the combination of an organochalcogen group with nitrone and oxime in the same molecule increases the antibacterial and antioxidant capabilities of the precursor (*R*)-citronellal. The synthesized compounds were effective in controlling foodborne pathogenic bacteria, including Gram-positive *L. monocytogenes*, *S. aureus*, and *B. cereus*; and Gram-negative *S.* Typhimurium and *E. coli* O157:H7, as well as the clinically important bacteria *P. aeruginosa*. Promising results on the antimicrobial activity were obtained in the disk diffusion test. Nitrone **5a**, with a phenylthio group at the α-position, presented the lowest MIC, for *B. cereus* (0.48 mM), while the lowest MBC was observed using compound **5c** against *E. coli* O157:H7 (0.52 mM). Regarding the antioxidant activities, all of the synthesized compounds were active. Compounds **5c** (sulfur-containing), oxime **10** (selenium-containing) and nitrone **5d** (without chalcogen) presented the best results in DPPH, ABTS, and FRAP assays, respectively. Among the five tested compounds, only α-phenylselanyl nitrone **5b** showed acute toxicity, reducing the δ-ALA-D activity in liver and kidney of mice. The in vitro data presented here indicate that thio- and selenium-containing nitrones and oxime derivatives from (*R*)-citronellal have potential to be explored in the food industry to enhance the safety of food products, protecting from oxidation and foodborne bacteria. Additional studies on the long-term toxicity need to be performed before a possible commercial utilization.

## Figures and Tables

**Table 1:** Antimicrobial activity of compounds **5a–d** and **10** in the agar disk diffusion test.

Bacteria **	Inhibition Zone * (mm) Compounds						
	Streptomycin (10 µg)	5a	5b	5c	5d	10	9e
*L. monocytogenes*	21	18	19.5	26	31	21	21
*S. aureus*	21	15	19.5	24	24.5	22	22
*B. cereus*	26	21.5	18.5	24.5	29	19	19
*S.* Typhimurium	18	8.5	8.5	21.5	23	10.5	10.5
*P. aeruginosa*	13	8.5	8.5	24	23	9	9
*E. coli* O157:H7	24	11	9	21.5	22.5	9	9

Notes: * Results are the mean of two repetitions; ** Bacteria inoculated at the concentration of 1.0 × 10^8^ CFU.mL^−1^.

**Table 2:** Minimum inhibitory concentration (MIC) and minimum bactericidal concentration (MBC) of compounds **5a–d** and **10**.

Compound	Bacteria **											
	*L. monocytogenes*		*S. mureus*		*B. mereus*		*S.* Typhimurium		*P. aeruginosa*		*E. coli* O157:H7	
	MIC ^a,^*	MBC ^a,^*	MIC ^a,^*	MBC ^a,^*	MIC ^a,^*	MBC ^a,^*	MIC ^a,^*	MBC ^a,^*	MIC ^a,^*	MBC ^a,^*	MIC ^a,^*	MBC ^a,^*
**5a**	>0.65	>0.65	>0.65	>0.65	0.48	0.59	>0.65	>0.65	>0.65	>0.65	>0.65	>0.65
**5b**	>0.64	>0.64	0.64	>0.64	0.64	>0.64	>0.64	>0.64	>0.64	>0.64	>0.64	>0.64
**5c**	0.69	>0.69	0.61	0.69	0.69	0.69	>0.69	>0.69	0.61	0.61	0.52	0.52
**5d**	1.26	1.26	1.26	1.26	>1.26	>1.26	1.13	1.26	1.13	1.26	1.13	1.26
**10**	>0.70	>0.70	>0.70	>0.70	>0.70	>0.70	>0.70	>0.70	>0.70	>0.70	>0.70	>0.70

Notes: ^a^ Values in mM.mL^−1^; * Results are the mean of two repetitions; ** Bacteria inoculated at the concentration of 10^5^ CFU.mL^−1^.

**Table 3:** DPPH radical scavenging of compounds **5a** and **5c**.

Concentration (μM)	Compounds	
	5a	5c
100	2.43 ± 1.08	2.43 ± 0.61
500	20.31 ± 1.26 ***	29.34 ± 1.00 ***

Notes: Data are represented as mean ± SD (*n* = 3). The values are expressed in percentage of inhibition in relation to control. The asterisks represent significant difference (***) *p* < 0.001 when compared with control sample by Student–Newman–Keuls test for post-hoc comparison.

**Table 4:** ABTS radical scavenging of compounds **5a–d** and **10**.

Concentration (μM)	Compounds				
	5a	5b	5c	5d	10
5	nt	nt	nt	nt	2.39 ± 0.57
10	-	-	-	-	27.81 ± 2.12 ***
50	11.74 ± 0.47 ***	9.69 ± 0.57 **	4.18 ± 1.23	0.30 ± 0.33	87.59 ± 4.81 ***
100	22.84 ± 1.02 ***	22.32 ± 1.65 ***	11.60 ± 1.50 ***	3.10 ± 0.48 ***	94.03 ± 1.05 ***
500	77.61 ± 0.81 ***	73.92 ± 4.96 **	59.51 ± 4.16 ***	17.05 ± 0.47 ***	*x*
IC_50_	297.00 ± 1.00	315.30 ± 30.02	419.30 ± 35.80	-	25.00 ± 2.00

Notes: Data are represented as mean ± SD (*n* = 3). The values are expressed in percentage of inhibition in relation to control. IC_50_ = concentration compound required for 50% scavenging, in μM. The asterisks represent significant difference (**) *p* < 0.01; (***) *p* < 0.001 when compared with control sample by Student–Newman–Keuls test for post-hoc comparison. nt = not tested. *x* = sample blurred color.

**Table 5:** Ferric ion reducing antioxidant power (FRAP) of compounds **5a–d** and **10**.

Concentration (μM)	Compounds				
	5a	5b	5c	5d	10
Control	0.118 ± 0.009	0.094 ± 0.026	0.071 ± 0.020	0.117 ± 0.009	0.071 ± 0.020
1	-	nt	nt	0.166 ± 0.008	nt
5	0.165 ± 0.007	-	-	0.280 ± 0.022 **	-
10	0.207 ± 0.019 *	0.133 ± 0.097	0.153 ± 0.047	0.412 ± 0.055 ***	0.084 ± 0.010
50	0.572 ± 0.023 ***	0.256 ± 0.181	0.543 ± 0.047 ***	1.198 ± 0.024 ***	0.180 ± 0.008 ***
100	1.039 ± 0.067 ***	0.416 ± 0.234	1.121 ± 0.160 ***	*x*	0.257 ± 0.007 ***
500	*x*	1.602 ± 0.408 ***	*x*	*x*	1.291 ± 0.045 ***

Notes: Data expressed mean ± SD (*n* = 3). The asterisks represent significant difference (*) *p* < 0.05; (**) *p* < 0.01; (***) *p* < 0.001 when compared with control sample (FRAP solution without compounds) by Student–Newman-Keuls test for post-hoc comparison. nt = not tested. *x* = absorbance above the spectrophotometer limit.

**Table 6:** Assay of δ-ALA-D in liver, kidney, and brain of rats after treatment with compounds **5a–d** and **10**.

Concentration (μM)	Compounds				
	5a	5b	5c	5d	10
*Liver*					
control	4,79 ± 0.87	4.79 ± 0.87	4.79 ± 0.87	4.30 ± 0.74	4.30 ± 0.74
10	4.99 ± 0.84	2.59 ± 0.29 ***	4.63 ± 0.82	4.50 ± 0.60	4.90 ± 0.58
50	4.87 ± 0.67	0.42 ± 0.13 ***	4.31 ± 0.81	4.22 ± 0.78	4.71 ± 0.53
100	4.71 ± 0.89	0.07 ± 0.02 ***	4.12 ± 0.90	3.54 ± 0.84	4.57 ± 0.62
500	4.46 ± 0.80	0.03 ± 0.01 ***	3.88 ± 0.87	2.84 ± 0.57	3.97 ± 0.82
*Kidney*					
control	1.01 ± 0.12	1.01 ± 0.12	1.01 ± 0.12	0.98 ± 0.11	0.98 ± 0.11
10	1.17 ± 0.05	1.08 ± 0.05	1.10 ± 0.12	1.02 ± 0.11	0.96 ± 0.12
50	1.17 ± 0.07	0.99 ± 0.07	1.02 ± 0.12	0.96 ± 0.12	0.95 ± 0.10
100	1.09 ± 0.08	0.68 ± 0.12 **	0.99 ± 0.10	0.89 ± 0.12	0.84 ± 0.10
500	0.90 ± 0.08	0.24 ± 0.03 ***	0.90 ± 0.07	0.72 ± 0.07	0.61 ± 0.12 *
*Brain*					
control	0.30 ± 0.05	0.26 ± 0.05	0.30 ± 0.05	0.30 ± 0.05	0.30 ± 0.05
10	0.44 ± 0.09	0.30 ± 0.11	0.30 ± 0.10	0.29 ± 0.09	0.30 ± 0.10
50	0.42 ± 0.10	0.25 ± 0.05	0.28 ± 0.09	0.27 ± 0.10	0.28 ± 0.09
100	0.40 ± 0.11	0.18 ± 0.03	0.26 ± 0.08	0.26 ± 0.09	0.26 ± 0.08
500	0.36 ± 0.10	0.14 ± 0.04	0.26 ± 0.07	0.24 ± 0.08	0.26 ± 0.07

Notes: Data expressed mean ± SD (*n* = 3). The asterisks represent significant difference (**) *p* < 0.01; (***) *p* < 0.001 when compared with control sample by Student–Newman–Keuls test for post-hoc comparison.

## Supplementary Materials

The following are available online at www.mdpi.com/2305-6320/4/2/39/s1, Figure S1: ^1^H NMR of compound **5a** in CDCl_3_ (300 MHz), Figure S2: ^13^C NMR of compound **5a** in CDCl_3_ (75 MHz), Figure S3: ^1^H NMR of compound **5b** in CDCl_3_ (300 MHz), Figure S4: ^13^C NMR of compound **5b** in CDCl_3_ (75 MHz), Figure S5: ^1^H NMR of compound **5c** in CDCl_3_ (300 MHz), Figure S6: ^13^C NMR of compound **5c** in CDCl_3_ (75 MHz), Figure S7: ^1^H NMR of compound **5d** in CDCl_3_ (300 MHz), Figure S8: ^13^C NMR of compound **5d** in CDCl_3_ (75 MHz), Figure S9: ^1^H NMR of compound **10** in CDCl_3_ (400 MHz), Figure S10: ^13^C NMR of compound **10** in CDCl_3_ (100 MHz).
