# Supplementary material for: Synthesis, Antimicrobial, and Antioxidant Activities of Chalcogen-Containing Nitrone Derivatives from (R)-citronellal
Source: Medicines (Basel). 2017 Jun 10;4(2):39. doi: 10.3390/medicines4020039 (PMC5590075; doi:10.3390/medicines4020039)
Supplement: Supplementary file 1 [file medicines-04-00039-s001.pdf]

Supplementary Materials: Synthesis, Antimicrobial, and Antioxidant Activities of Chalcogen-Containing Nitrone Derivatives from (*R*)-citronellal

Mariana C. Ferraz, Renata A. Mano, Daniela H. Oliveira, Darla S. V. Maia, Wladimir P. Silva, Lucielli Savegnago, Eder J. Lenardão and Raquel G. Jacob

Experimental Section

General Information: The reactions were monitored by TLC carried out on Merck silica gel (60 F254) by using UV light as visualizant agent and 5% vanillin in 10% H_2SO_4 and heat as developing agents. Baker silica gel (particle size 0.040–0.063 mm) was used for flash chromatography. Proton nuclear magnetic resonance spectra (^{1}H NMR) were obtained at 300 MHz on a Varian Gemini NMR and at 400 MHz on Bruker DPX 400 spectrometer. Spectra were recorded in $CDCl_3$ solutions. Chemical shifts are reported in ppm, referenced to tetramethylsilane (TMS) as the external reference. Coupling constants (J) are reported in Hertz. Abbreviations to denote the multiplicity of a particular signal are s (singlet), d (doublet), t (triplet), q (quartet) and m (multiplet). Carbon-13 nuclear magnetic resonance spectra (^{13}C NMR) were obtained at 75 MHz on a Varian Gemini NMR and at 100 MHz on Bruker DPX 400 spectrometers. Chemical shifts are reported in ppm, referenced to the solvent peak of $CDCl_3$. Low-resolution mass spectra were obtained with a Shimadzu GC-MS-QP2010 mass spectrometer.

1. Synthesis of compounds, biological activities, spectral and analytical data

*1.1. Synthesis of α-phenylselanyl citronellal **3a**, α-phenylthio citronellal **3b** and β-phenylthio citronellal 8*

The synthesis of α-phenylchalcogen citronellal **3a–b** was performed according to the methodology developed by Nazari and Movassagh [1], with modifications. In a 25 mL vial was added (R)-citronellal (**1**, 0.308 g, 2 mmol), diphenyl disulfide (**2a**, 1.5 mmol) or diphenyl diselenide (**2b**, 2 mmol) and PEG-400 (4.0 mL) under N_2 atmosphere. Then, Al_2O_3/KF 40% (0.324 g, 1.5 mmol) was added and the temperature was slowly increased to 60°C. The progress of the reaction was monitored using thin layer chromatography (TLC) and after 22 h, compounds 1a-b were isolated and identified. The synthesis of β-phenylchalcogen citronellal **8** was performed according to previously described by our group [2]. In a test tube was added citral (**6**, 0.304 g, 2 mmol), benzenethiol (**7**, 0.352 g, 2.4 mmol) and Al_2O_3/KF 40% (0.140 g, 0.65 mmol) under magnetic stirring at room temperature. The progress of the reaction was monitored using thin layer chromatography (TLC) and after 24 h, compound 8 was isolated.

*1.2. General procedure for the synthesis of nitrones **5a-d** derived from citronellal*

Using a synthetic route adapted from Isager *et al.*[3] in a 25 mL vial was added the aldehyde 1, **3a–b** or 8 (0.5 mmol), N-methyl-hydroxylamine hydrochloride (**4**, 0.084 g, 1 mmol) and water (2 mL) as the solvent and the mixture was stirred at room temperature for 30 min. Then, a 1M solution of Na_2CO_3 (1.0 ml) was added and the stirring was continued for additional 24 h. Compound **5a** was purified by preparative chromatographic plate (silicagel) and compounds **5b-d** were isolated by column chromatography using neutral alumina as a stationary phase and a solution of hexanes/ethyl acetate as the eluent (90:10). The NMR spectra of nitrones **5a** (Figure S1 and Figure S2), **5b** (Figure S3 and Figure S4), **5c** (Figure S5 and Figure S6) and **5d** (Figure S7 and Figure S8) are in accordance with those expected for the compounds.

2. Synthesis of the selenium-containing oxime 10

Using a synthetic route adapted from Isager *et al.* [3], in a 25 mL vial was added α-phenylseleno citronellal (**5b**, 0.156 g, 0.5 mmol), hydroxylamine hydrochloride (**9**, 0.069 g, 1 mmol) and water (2

mL) as the solvent. After stirring for 30 min at room temperature, it was added 0.5 mL of an aqueous solution of Na_2CO_3 (0.027 g, 0.26 mmol) and the stirring was continued for additional 22 h. After this time, the oxime **10** was isolated by column chromatography using silicagel as a stationary phase and a solution of hexanes/ethyl acetate (95:5) as the eluent. The NMR spectra of oxime **10** (Figure S9 and Figure S10) are in accordance with those expected for the compound.

2.1. Antimicrobial activity assay using the disk diffusion test

The disk diffusion test followed the methodology recommended by the Clinical Laboratory Standards Institute CLSI [4]. The inoculum was standardized by standard McFarland to the concentration of 10^8 $CFU.mL^{-1}$ and spread on the surface of a Petri dish containing Mueller-Hinton (MH) agar. Then, the paper disc (6 mm) was put on a plate impregnated with 20 μL of the testing compound previously diluted in DMSO (1:1) and incubated at 37 °C for 24 hours. Zone of inhibition ≥20 mm were considered as strong inhibition, <20–12 mm as moderate inhibition, and <12 mm no inhibition. A positive control with streptomycin antibiotic (10 μg) and a negative one, with paper discs impregnated with distilled water, were used

2.2. Determination of the minimum inhibitory concentration (MIC) and minimum bactericidal concentration (MBC)

The MIC was determined using the macro dilution tube method, in accordance with Rota *et al.* [5], with modifications. The tested concentrations were 20, 18, 15, 12, 10, 7 and 5 $\mu L.mL^{-1}$ of each compound. In a tube with 1 mL of MH broth, was inoculated 10^5 $UFC.mL^{-1}$, the compound in a predetermined concentration and, to facilitate the solubility, 2 drops of TWEEN 80. The mixture was incubated at 37 °C for 24 h under stirring (150 rpm). As the positive control, MH broth and inoculum concentration in 10^5 $UFC.mL^{-1}$ and as the negative control MH, the inoculum concentration in 10^5 $UFC{\cdot}mL^{-1}$ and streptomycin (10 μg) were used. MIC was defined as the lowest concentration of compound which showed no visible growth in broth. After 24 h, tubes with no visible growth were seeded in a 100 mL TSA-YE tube and incubated at 37 °C for 24 h. The MBC is the lowest concentration of compound where 99.9% of the initially inoculated cells were killed.

2.3. Antioxidant activity assays

The antioxidant properties of the synthesized compounds were evaluated by three different methods *in vitro*: DPPH and $ABTS^+$ radical scavenging activity and ferric ion reducing antioxidant power (FRAP). All drugs were dissolved in dimethyl sulfoxide (DMSO). The experimental results were given as the means ± standard deviation (SD) to show the variations among the groups. The statistical analysis was performed using one-way analysis of variance (ANOVA) followed by Newman-Keuls multiple comparison test when appropriate. The differences were considered statistically significant at a probability of less than 5% ($p < 0.05$). All tests were performed at least three times in duplicate. The IC_{50} values (the concentration of sample required to scavenge 50% of the free radicals) were calculated from the graph of the scavenging effect percentage versus the compound concentration.

2.4. Radical Scavenging Activity

To determine if compounds **5a–d** and **10** present *in vitro* antioxidant activity against free radicals, DPPH and $ABTS^+$ scavenging capability were evaluated at different concentrations.

The DPPH scavenging activity of compounds **5a–d** and **10** (10–500 μM) was determined in accordance with the method of Sharma and Bhat [6] and the decrease in the absorbance at 517 nm was recorded.

The ABTS radical scavenging activity was determined according to the method described by Erel [7]. Different concentrations of compounds **5a–d** and **10** (10–500 μM) were mixed with the $ABTS^+$ solution, and the decrease in the absorbance at 734 nm was recorded.

The values are expressed as the percentages of radical inhibition (I %) in relation to the control values, as calculated by the following equation:

$$I\% = [(A_c – A_s/A_c) \times 100] \quad (1)$$

Where A_c is the absorbance of the control excluding the test compounds, and A_s is the absorbance of the tested compounds.

3. Ferric ion reducing antioxidant power (FRAP)

The ferric ions (Fe^{3+}) reducing antioxidant power (FRAP) method was used to measure the reducing capacity of compounds **5a–d** and **10**. The assay was performed as described by Stratil *et al.* [8] with slight modifications. Different concentrations of **5a–d** and **10** (1–500 μM) and FRAP reagent were added to each sample, and the mixture was incubated at 37 °C for 40 min in the dark. The absorbance of the resulting solution was measured at 593 nm with a spectrophotometer.

In vitro toxicity

The activity of δ-ALA-D in the presence of compounds **5a–d** and **10** at different concentrations (10–500 μM) was determined according to the method described by Sassa [9]. Tissues were removed from the mice, liver and kidneys (1:10 *w*/*v*) and brain (1:5 *w*/*v*). They were homogenized in a 0.1 M Tris-HCl buffer, pH 7.4 and centrifuged at 2500 RPM for 10 min. The supernatants were used for determination of the δ-ALAD activity.

Firstly, 200 μL of the tissue supernatant was incubated for 10 min at 37 °C with 50 μL of potassium phosphate buffer (TFK), 150 μL of distilled water, and 10 μL of the test compound. Then, 100 μL of substrate (12 mM ALA aminolevulinic acid) was added and the incubation was carried out at 37 °C for 1 h for liver and kidney and 3 h for the brain; the reaction was stopped by adding 250 μL of trichloroacetic acid (TCA) solution with 10% 10 mM $HgCl_2$ followed by centrifugation (2500 RPM for 10 min). Then, 500 μL of the supernatant was collected and added to 1000 μL of Ehrlich's reagent and 500 μL of distilled water. The resulting mixture was incubated for 10 min at room temperature and the absorbance was read at 555 nm.

The results were expressed in nmol PBG.mg.tn^{-1}.h^{-1}, according to the equation below:

$$PBG = [(2\ nmol/0.061)\ (1/T) \times (1/ptn) \times (1/tissue) \times Abs] \quad (2)$$

Where T is the incubation time, ptn is protein and tissue refers to the added amount, for example 0.2 to 200 μL of tissue and Abs refers to the absorbance of the tested compounds.

(3*R*,*E*)-*N*-3,7-trimethyl-2-(phenylthio)oct-6-en-1-imine oxide (**5a**)

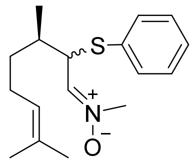

Yield: 61%; yellow oil. ^{1}H NMR ($CDCl_3$, 300 MHz): δ = 7.40–7.37 (m, 2H), 7.30–7.25 (m, 2H), 7.21 –7.16 (m, 1H), 6.72–6.67 (m, 1H), 5.10–5.05 (m, 1H), 4.76–4.67 (m, 1H), 3.61 (s, 3H), 2.09 – 1.94 (m, 3H), 1.68 (s, 3H), 1.59 (s, 3H), 1.38–1.25 (m, 2H), 1.09 (t, J = 6.56 Hz, 3H). ^{13}C NMR ($CDCl_3$, 75 MHz): δ = 140.0, 139.2, 134.5, 131.9, 130.1, 130.1, 128.9, 126.5, 123.8, 52.6, 52.6, 48.4, 47.6, 35.9, 35.6, 34.5, 34.2, 25.7, 25.6, 25.4, 17.7, 17.6, 17.2, 16.6. MS, m/z(rel. int.): 291[M]+(12), 182(34), 135(21), 123(18), 84(27), 69(66), 41(100).

(3*R*,*E*)-*N*-3,7-trimethyl-2-(phenylselanyl)oct-6-en-1-imine oxide (**5b**)

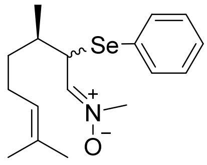

Yield: 65%; yellow oil. ^{1}H NMR ($CDCl_3$, 300 MHz): δ = 7.65–7.60 (m, 2H), 7.30–7.24 (m, 3H), 6.74

(dd, *J* = 2.57 Hz and 9.14 Hz, 1H), 5.07–5.00 (m, 1H), 4.70–4.62 (m, 1H), 3.56 (d, *J* = 4.50 Hz, 3H), 2.07–1.79 (m, 3H), 1.66 (s, 3H), 1.58 (d, *J* = 6.40 Hz, 3H), 1.35–1.23 (m, 2H), 1.07 (dd, *J* = 6.72 Hz and 14.83 Hz, 3H). ^{13}C NMR (CDCl$_3$, 75 MHz): δ = 139.6, 139.0, 134.8, 134.7, 131.7, 128.8, 128.7, 128.1, 128.0, 127.6, 123.7, 52.3, 52.3, 45.3, 44.3, 35.5, 35.5, 35.1, 34.8, 25.6, 25.4, 25.1, 17.9, 17.5, 17.5, 17.2. MS, m/z(rel. int.): 339[M]+(4), 182(99), 156(18), 135(14), 109(27), 84(31), 77(32), 69(61), 41(100).

(E)- and *(Z)*-*N*-3,7-trimethyl-3-(phenylthio)oct-6-en-1-imine oxide (**5c**)

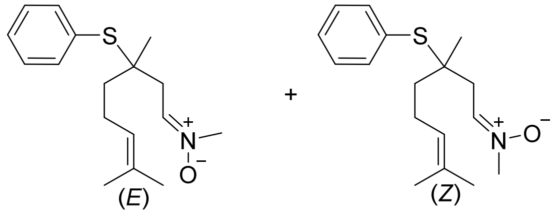

Yield: 67%; yellow oil. ^{1}H NMR (CDCl$_3$, 300 MHz): δ = 7.51–7.19 (m, 5H), 7.05–7.02 (m, 0.6H), 6.60–6.57 (m, 0.4H), 5.10–5.02 (m, 1H), 3.75 (s, 1.8H), 3.71 (s, 1.2H), 2.65 (d, *J* = 5.42 Hz, 1H), 2.23–2.12 (m, 3H), 1.83 (s, 1H), 1.68 (s, 3H), 1.62 (s, 1.8H), 1.61 (s, 1.2H), 1.55–1.49 (m, 1H), 1.26 (s, 3H). ^{13}C NMR (CDCl$_3$, 75 MHz): δ = 137.7, 137.3, 132.3, 132.1, 130.6, 129.1, 129.0, 128.8, 123.4, 123.1, 122.9, 116.6, 115.8, 52.6, 52.2, 52.2, 50.6, 40.5, 40.3, 37.2, 33.5, 26.5, 26.2, 25.9, 25.6, 24.4, 23.0, 17.9, 17.7. MS, m/z(rel. int.): 291[M]+(1), 181(10), 135(17), 110(20), 98(100), 81(13), 69(64), 55(14), 41(75).

(*R*,*E*)-*N*-3,7-trimethyloct-6-en-1-imine oxide (**5d**)

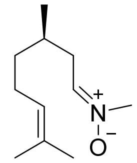

Yield: 61%; yellow oil. ^{1}H NMR (CDCl$_3$, 300 MHz): δ = 6.73–6.69 (m, 1H), 5.10–5.04 (m, 1H), 3.70 (s, 3H), 2.75–2.67 (m, 1H), 2.56–2.31 (m, 2H), 2.05–1.72 (m, 3H), 1.68 (s, 3H), 1.60 (s, 3H), 1.43–1.19 (m, 2H), 0.96 (d, *J* = 6.68 Hz, 3H). ^{13}C NMR (CDCl$_3$, 75 MHz): δ = 139.9, 131.5, 124.0, 52.3, 36.8, 33.9, 30.1, 25.6, 25.3, 19.8, 17.5. MS, m/z(rel. int.): 183[M]+(19), 137(20), 109(31), 100(70), 81(37), 69(69), 55(36), 41(100).

(3*R*,*E*)- and (3*R*,*Z*)-3,7-dimethyl-2-(phenylselanyl)oct-6-enal oxime (**10**)

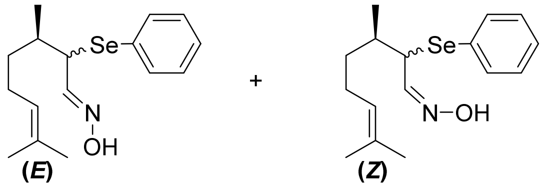

Yield: 90%; yellow oil. ^{1}H NMR (CDCl$_3$, 400 MHz): δ = 8.98 (brs, 0.5H), 8.65 (brs, 0.5H), 7.57 – 7.48 (m, 2H), 7.44–7.36 (m, 0.5H), 7.25–7.17 (m, 3H), 6.79–6.77 (m, 0.5H), 5.11–5.04 (m, 1H), 4.66–4.57 (m, 0.5H), 3.80–3.73 (m, 0.5H), 2.38–1.76 (m, 3H), 1.67 (s, 3H), 1.59 (s, 3H), 1.42–1.16 (m, 2H), 1.11–0.91 (m, 3H). ^{13}C NMR (CDCl$_3$, 100 MHz): δ = 151.6, 151.3, 151.2, 150.6, 150.5, 135.4, 135.3, 135.0, 134.9, 131.8, 131.7, 131.6, 131.3, 128.9, 128.8, 128.3, 128.2, 127.8, 127.6, 124.3, 124.3, 124.0, 123.9, 123.8, 50.2, 49.0, 42.9, 41.7, 36.8, 36.6, 36.3, 35.7, 35.4, 35.3, 35.2, 34.7, 34.6, 31.9, 30.9, 30.5, 25.6, 25.5, 25.4, 25.3, 25.2, 19.7, 19.4, 17.9, 17.6, 17.5, 17.1, 17.0. MS, m/z(rel. int.): 325[M]+(1), 308(4), 168(63), 157(23), 135(9), 123(22), 109(21), 77(39), 69(97), 41(100).

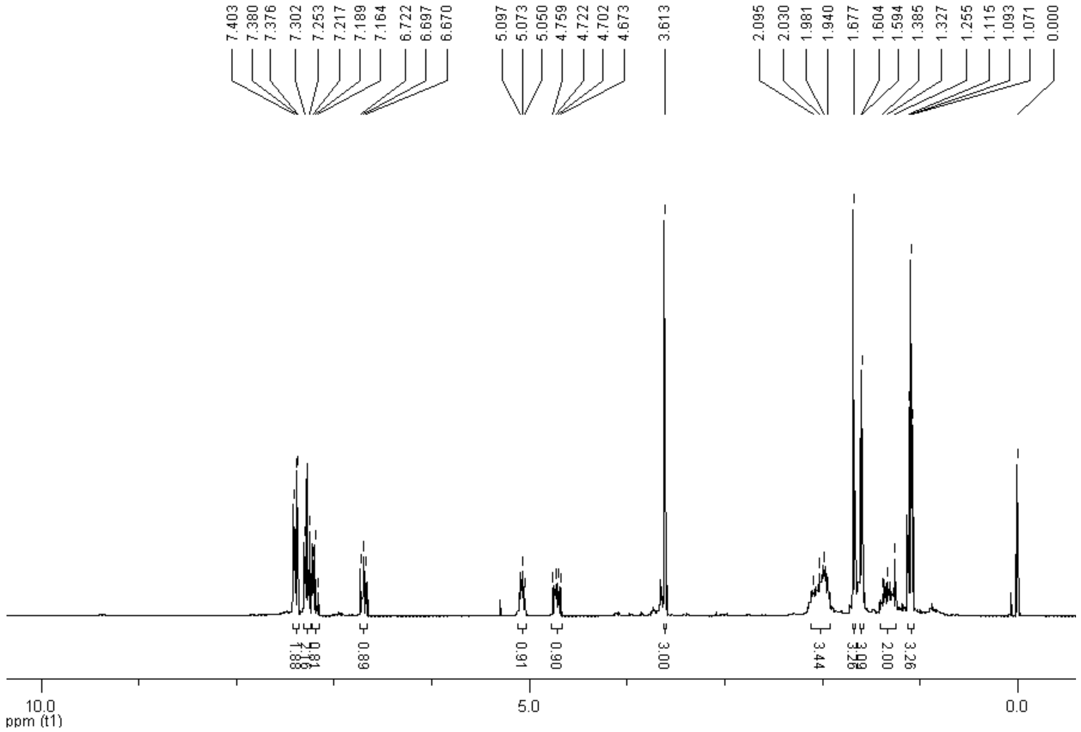

Figure S1. ^{1}H NMR of compound **5a** in $CDCl_3$ (300 MHz).

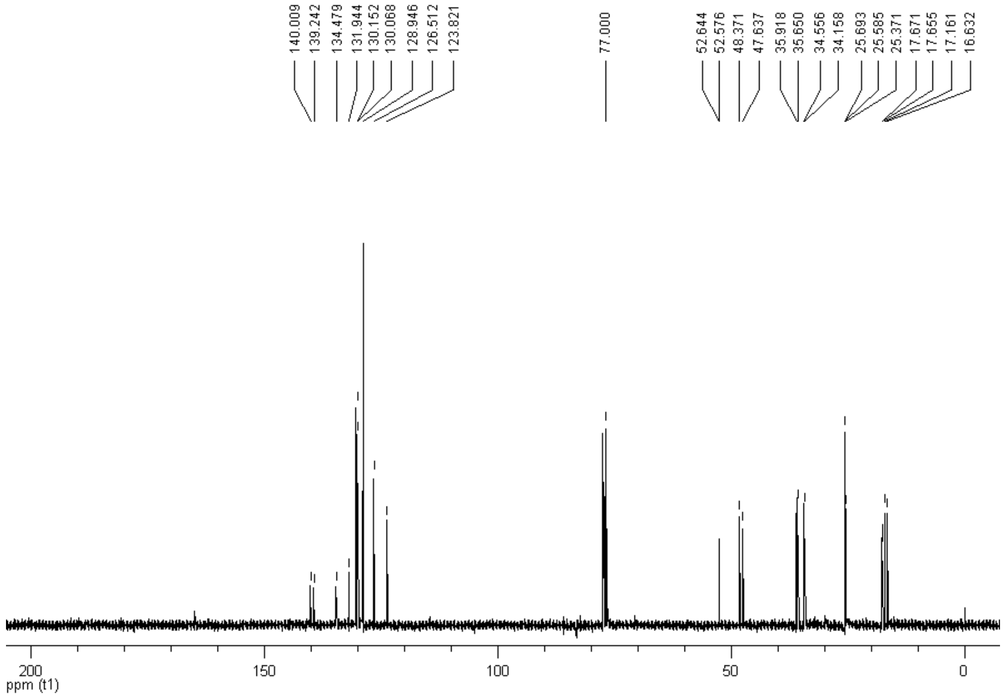

Figure S2. ^{13}C NMR of compound **5a** in $CDCl_3$ (75 MHz).

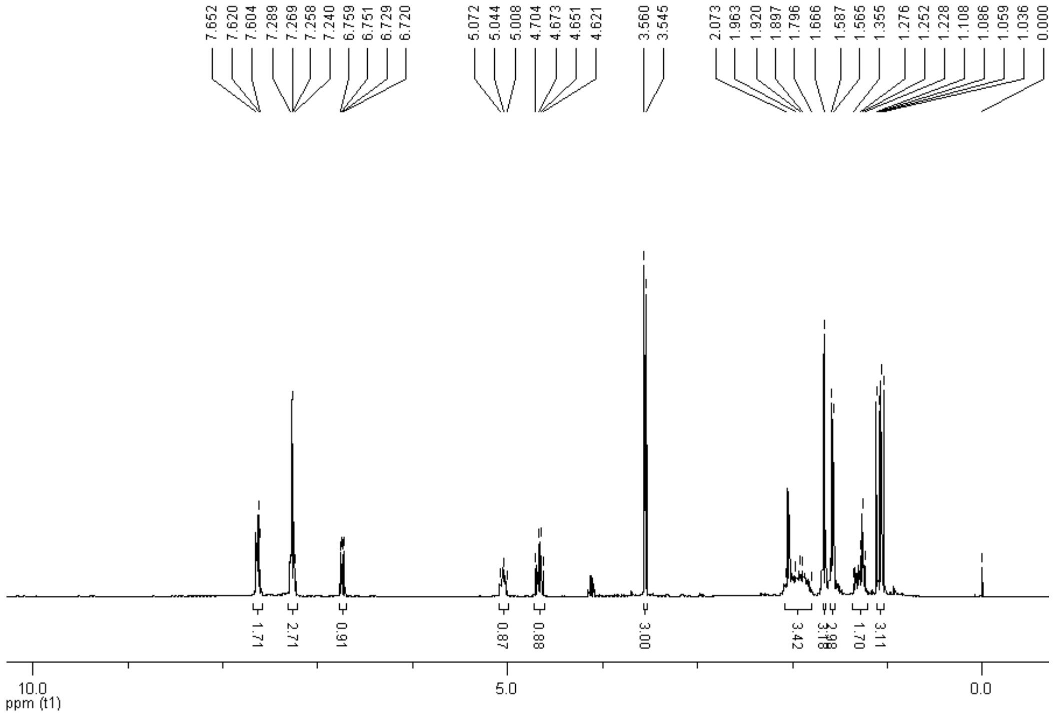

Figure S3. ^{1}H NMR of compound **5b** in $CDCl_3$ (300 MHz).

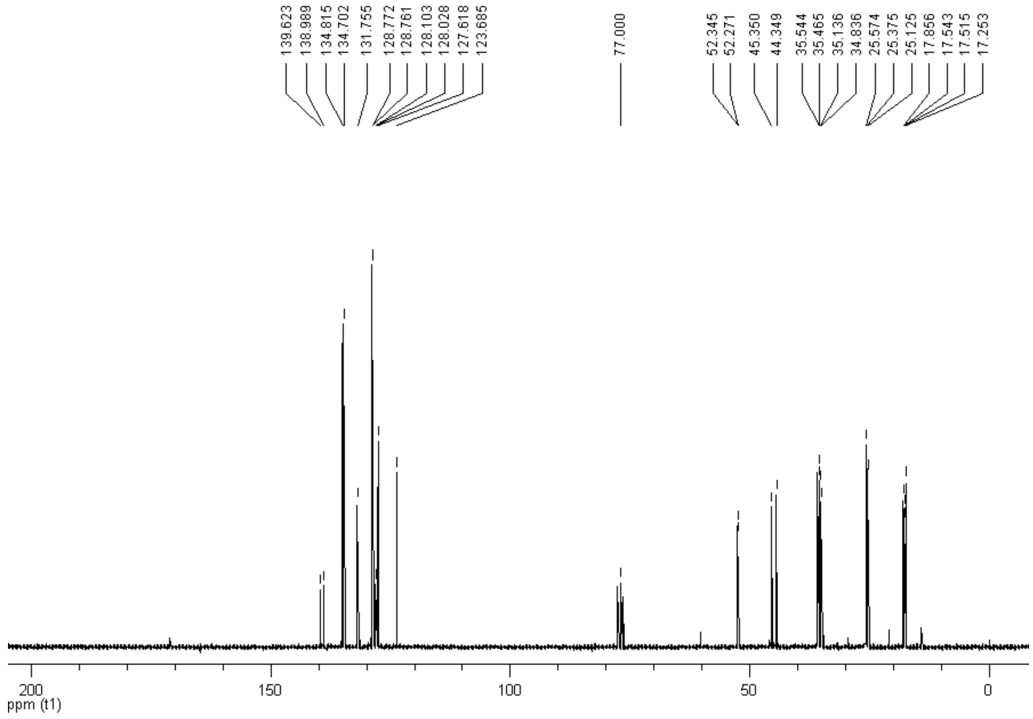

Figure S4. ^{13}C NMR of compound **5b** in $CDCl_3$ (75 MHz).

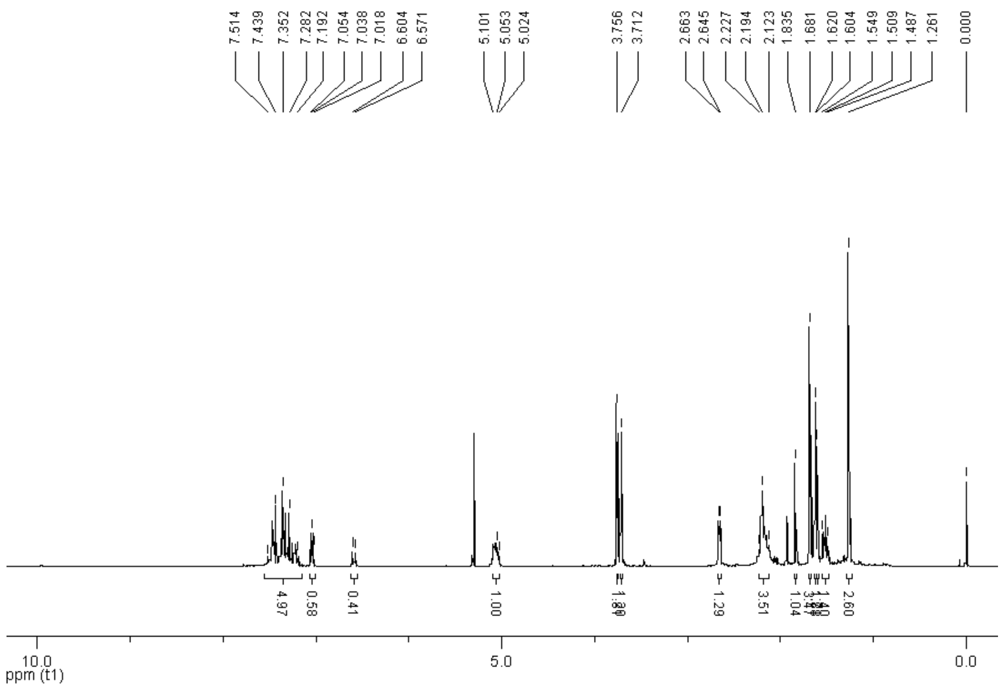

Figure S5. ^{1}H NMR of compound **5c** in $CDCl_3$ (300 MHz).

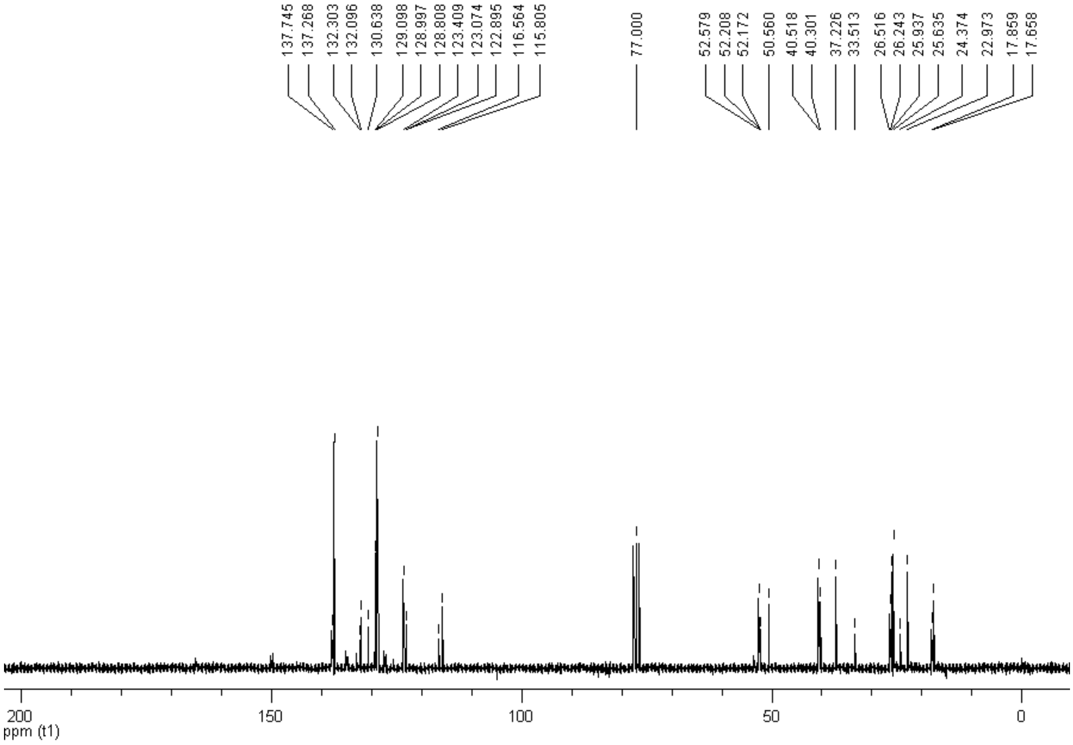

Figure S6. ^{13}C NMR of compound **5c** in $CDCl_3$ (75 MHz).

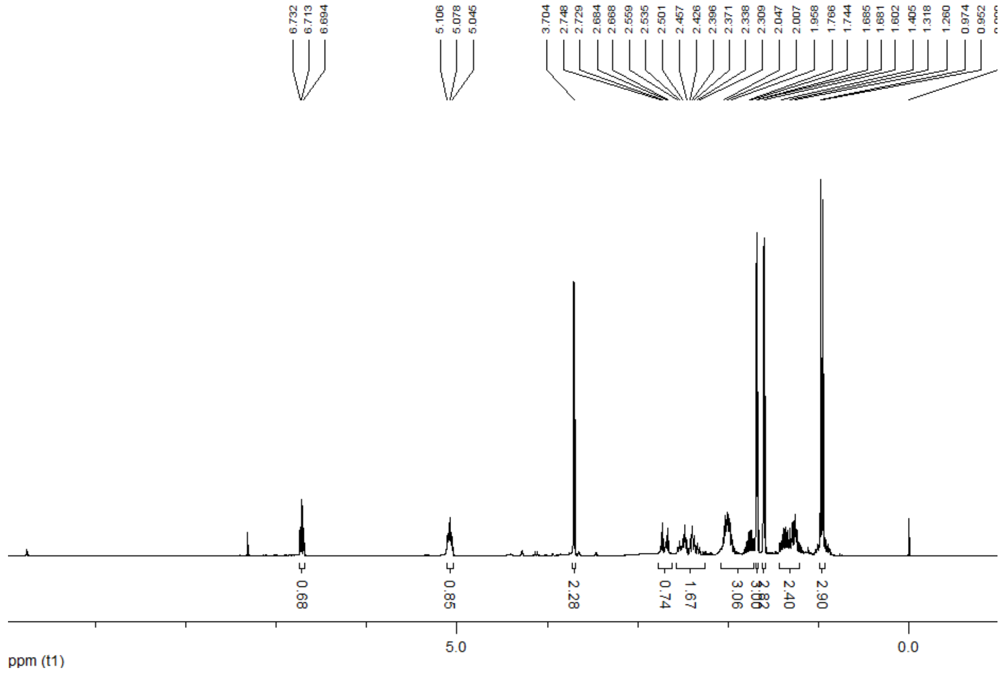

Figure S7. ^{1}H NMR of compound **5d** in $CDCl_3$ (300 MHz).

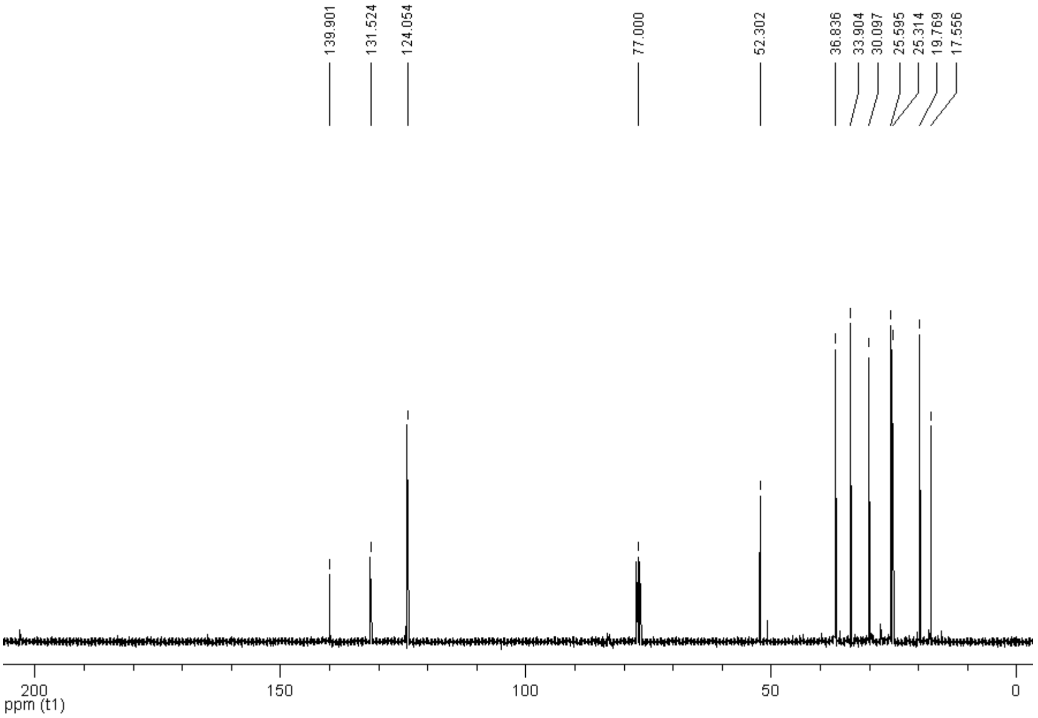

Figure S8. ^{13}C NMR of compound **5d** in $CDCl_3$ (75 MHz).

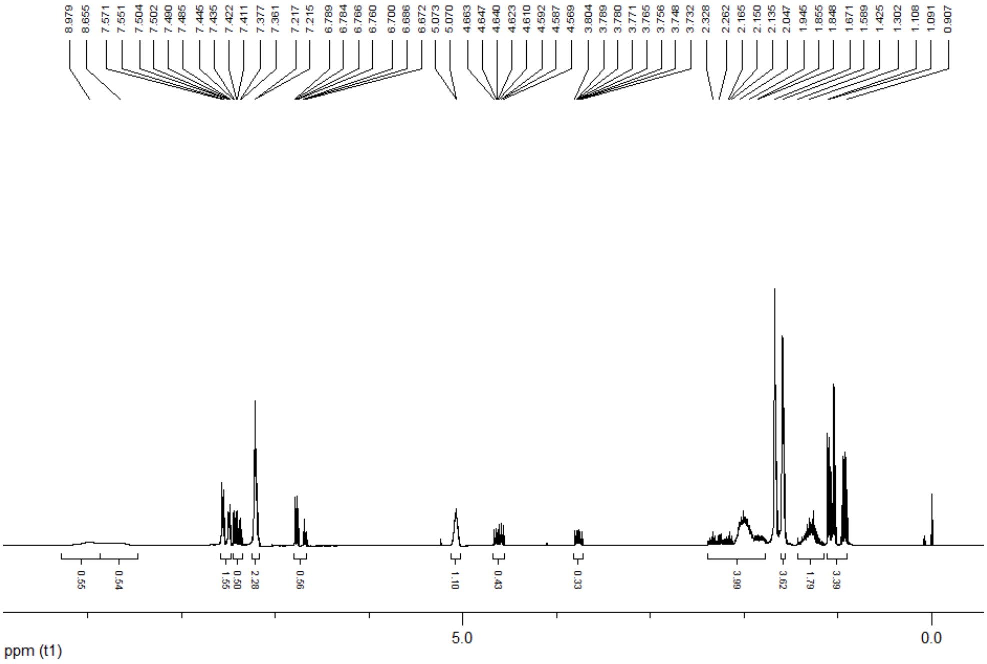

Figure S9. ^{1}H NMR of compound **10** in $CDCl_3$ (400 MHz).

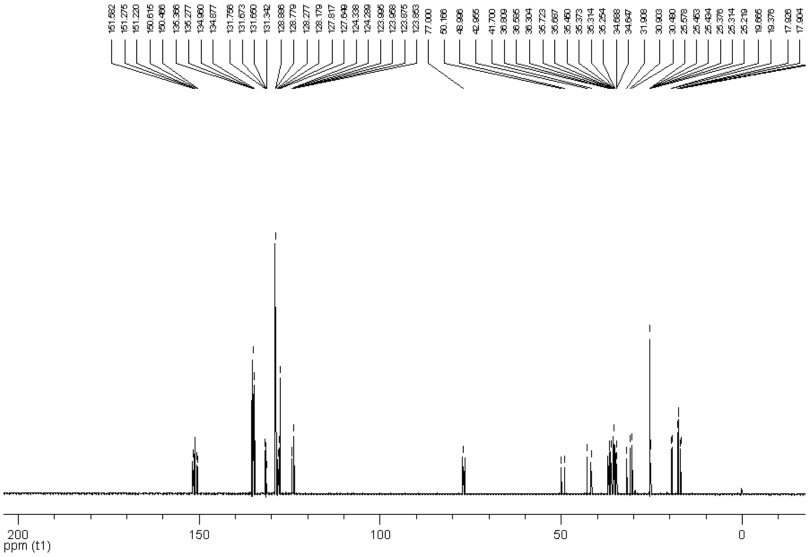

Figure S10. ^{13}C NMR of compound **10** in $CDCl_3$ (100 MHz).

References

1. Nazari, M.; Movassagh, B. α-Phenylselenenylation of aldehydes and ketones with diphenyl diselenide mediated by KF/Al2O3. *Tetrahedron Lett.* **2009**, *50*, 1453–1455. doi:10.1016/j.tetlet.2009.01.068.
2. Lenardão, E.J.; Trecha, D.O.; Ferreira, P.C.; Jacob, R.G.; Perin, G. Green Michael addition of thiols to electron deficient alkenes using KF/alumina and recyclable solvent or solvent-free conditions. *J. Braz. Chem. Soc.* **2009**, *20*, 93–99. doi:10.1590/S0103-50532009000100016.
3. Isager, P.; Thomsen, I.; Torssell, K.B.G. Reactions with α,β-unsaturated nitrile oxides. Synthetic studies in the terpene field. Synthesis of tangetones, ocimenones, deodarone and atlantone. *Acta Chem. Scand.* **1990**, *44*, 806–813. doi:10.3891/acta.chem.scand.44–0806.
4. CLSI-Clinical Laboratory Standards Institute. *Performance standards for antimicrobial susceptibility testing; Twenty-second informational supplement (M100-S25)*. Clinical Laboratory Standards Institute: Wayne, PA, USA, 2015.

5. Rota, C; Carramiñana, J.J.; Burillo, J.; Herrera, A. In vitro antimicrobial activity of essential oils from aromatic plants against selected foodborne pathogens. *J. Food Prot*. **2004**, *67*, 1252–1256. doi:10.4315/0362-028X-67.6.1252.
6. Sharma, O.P.; Bhat, T.K. DPPH antioxidant assay revisited. *Food Chem.* **2009**, *113*, 1201–1205. doi:10.1016/j.foodchem.2008.08.008.
7. Erel, O. A novel direct measurement method for total antioxidant capacity using a new generation, more stable ABTS radical cation. *Clin. Biochem.* **2004**, *37*, 277–285. doi:10.1016/j.clinbiochem.2003.11.015.
8. Stratil, P.; Klejdus, B.; Kuban, V. Determination of total content of phenolic compounds and their antioxidant activity in vegetables. Evaluation of spectrophotometric methods. *J. Agric. Food Chem.* **2006**, *54*, 607–616. doi:10.1021/jf052334j.
9. Sassa, S. Delta-aminolevulinic acid dehydratase assay. *Enzyme*. **1982**, *28*, 133–145. Available online: https://www.ncbi.nlm.nih.gov/pubmed/7140716 URL (accessed on 27 April 2017).
